# Survival analysis in breast cancer using proteomic data from four independent datasets

**DOI:** 10.1038/s41598-021-96340-5

**Published:** 2021-08-18

**Authors:** Ágnes Ősz, András Lánczky, Balázs Győrffy

**Affiliations:** 1 Department of Bioinformatics, Semmelweis University, Tűzoltó u. 7-9, 1094 Budapest, Hungary; 2 TTK Momentum Cancer Biomarker Research Group, Institute of Enzymology, 1117 Budapest, Hungary; 3 2nd Department of Pediatrics, Semmelweis University, 1094 Budapest, Hungary

**Keywords:** Cancer, Computational biology and bioinformatics

## Abstract

Breast cancer clinical treatment selection is based on the immunohistochemical determination of four protein biomarkers: ESR1, PGR, HER2, and MKI67. Our aim was to correlate immunohistochemical results to proteome-level technologies in measuring the expression of these markers. We also aimed to integrate available proteome-level breast cancer datasets to identify and validate new prognostic biomarker candidates. We searched studies involving breast cancer patient cohorts with published survival and proteomic information. Immunohistochemistry and proteomic technologies were compared using the Mann–Whitney test. Receiver operating characteristics (ROC) curves were generated to validate discriminative power. Cox regression and Kaplan–Meier survival analysis were calculated to assess prognostic power. False Discovery Rate was computed to correct for multiple hypothesis testing. We established a database integrating protein expression data and survival information from four independent cohorts for 1229 breast cancer patients. In all four studies combined, a total of 7342 unique proteins were identified, and 1417 of these were identified in at least three datasets. ESR1, PGR, and HER2 protein expression levels determined by RPPA or LC–MS/MS methods showed a significant correlation with the levels determined by immunohistochemistry (*p* < 0.0001). PGR and ESR1 levels showed a moderate correlation (correlation coefficient = 0.17, *p* = 0.0399). An additional panel of candidate proteins, including apoptosis-related proteins (BCL2,), adhesion markers (CDH1, CLDN3, CLDN7) and basal markers (cytokeratins), were validated as prognostic biomarkers. Finally, we expanded our previously established web tool designed to validate survival-associated biomarkers by including the proteomic datasets analyzed in this study (https://kmplot.com/). In summary, large proteomic studies now provide sufficient data enabling the validation and ranking of potential protein biomarkers.

## Introduction

Breast cancer is one of the most frequently diagnosed cancers and the leading cause of cancer-related death in women^1^. Routine utilization of histopathological markers has led to better survival outcomes in personalized therapy, while multigenic genomic and transcriptomic analyses have further defined clinically meaningful molecular subtypes^2^. Genomics provides the “blueprint” for cellular structure and functions, but due to its nature, it is always static, and the genome itself does not define the biological function. On the other hand, proteomics can show the physical structure of the cell, revealing a dynamic picture of active key functional elements. Proteomics can display the status of over 500,000 gene products defined by only approximately 30,000 genes^3^. Overall, proteomics can provide a snapshot of the biological functions within a cancer cell. However, the availability of clinically annotated proteomic data derived from large patient cohorts is still limited.

Routine methods used for protein quantification include antibody-based techniques, such as immunohistochemistry (IHC) and reverse-phase protein array (RPPA), enzyme-linked immunosorbent assays (ELISA) and mass spectrometry (MS)-based technologies. ELISA invented in the 1970s is extensively used in laboratory practice for analyzing a small number of proteins, but its limitations in multiplexing requiring high developmental costs and well-characterized antibodies prevented its large-scale application^4^. IHC is currently the gold standard method in routine pathological diagnosis, including the semiquantitative determination of Estrogen Receptor 1 (ESR1), Progesterone Receptor (PGR) and Human Epidermal Growth Factor Receptor 2 (HER2) receptor status in breast tumors. Is it possible to multiplex IHC using tissue microarrays, but these achieve higher output by simultaneously evaluating several patient samples and not by multiplexing the proteins simultaneously evaluated. Nevertheless, tissue microarrays play a solid role in uncovering new biomarkers in cancer research^5^. Although immunohistochemistry is the most frequently used protein analysis method in oncology, it has limits in the quantification and detection of activated proteins because the detection limit of IHC is often insufficient to measure phosphorylated proteins^6^.

In contrast to antibody-based methods, the RPPA technique, introduced in 2001, immobilizes the whole protein lysate on a solid phase in multiple dots^6^. A specific antibody solution is added to each array spot separately to achieve sensitive and simultaneous detection of proteins in small sample amounts (e.g., biopsy). RPPA requires well-specified antibodies, but it also makes it feasible to quantify the phosphorylation status of proteins and thus enables the characterization of entire pathways^7^.

Mass spectrometry (MS)-based technologies have rapidly advanced in recent years. In addition to speed, the second most prominent advantage of these methods is their ability to facilitate de novo identification and quantification of multiple proteins simultaneously. However, MS requires high initial cost, manual and time-consuming sample preparation, and an experienced technician to run the samples and interpret the data^8^. Three major quantitative MS-based techniques have been developed: directed, targeted, and shotgun (or discovery) proteomics^9^. In directed proteomics, a predefined set of peptide ions is quantified. In targeted proteomics, a set of predetermined fragment ions from anticipated, but not necessarily detected precursor ions is measured^9^. The shotgun method is based on the sequencing of peptides digested from the whole proteome and analyzing them via liquid chromatography and tandem mass spectrometry (LC‐MS/MS) and automated database searching^10^. Then, the protein quantity is calculated from the signal of detected peptides (ion intensity) or recorded number of MS/MS spectra (spectral counting). Protein abundance is normalized to the background proteome signal of measured samples (LFQ) or to an internal standard added to a labeled experiment^11,12^. Shotgun proteomics is superior to the other methods because it allows global and untargeted analysis of proteins thereby enabling better characterization of disease-associated changes at the protein level and the identification of new biomarkers.

RPPA, ELISA, and MS enable comprehensive large-scale analysis of the human proteome. International initiatives have emerged to facilitate collaboration and data sharing. The Human Proteome Organization (HUPO, www.hupo.org) initiated in 2010 the Human Proteome Project (HPP) aiming for the determination of the human proteome using a standardized analytical pipeline^13^. A major data repository for MS-based protein datasets is the ProteomeXchange Consortium (http://www.proteomexchange.org), which also includes PRIDE (http://www.ebi.ac.uk/pride), and PeptideAtlas (http://www.peptideatlas.org)^14^. The Human Protein Atlas portal (www.proteinatlas.org) provides antibody-based data of normal and cancerous tissues^15^. The Clinical Proteomic Tumor Analysis Consortium (CPTAC, https://cptac-data-portal.georgetown.edu/cptacPublic) of the National Cancer Institute curates combined genomic and proteomic data of multiple tumor types^16^. Finally, a side project of The Cancer Genome Atlas (TCGA) Project, The Cancer Proteome Atlas (TCPA, https://tcpaportal.org/tcpa/index.html) contains a large RPPA-based protein expression cohort^12^.

Breast cancer is classified into four molecular subtypes, each having different molecular and prognostic characteristics^17^. In the clinical routine, immunohistochemistry is used to measure the presence of estrogen receptor (ESR1), progesterone receptor (PGR), human epidermal growth factor receptor 2 (HER2) and the proliferation marker MKI67. Evaluation of these biomarkers is mandatory to assign patients into clinically effective treatment subtypes termed basal (receptor negative), luminal A (ESR1 and PGR positive and low MKI67), luminal B (ESR1 and PGR positive and high MKI67), and HER2-enriched (HER2 positive ESR1 negative)^18^. Of note, additional markers, including androgen receptor (AR), epidermal growth factor receptor (EGFR) and cytokeratins (CK), have also been proposed for biomarker-based subtyping^19,20^.

Proteomic datasets comprise a large amount of protein-level data for each included specimen, and therefore, these datasets can provide an opportunity to validate existing prognostic biomarkers. In addition, by simultaneously analyzing multiple proteins in the same sample cohort, one can compare and rank new biomarker candidates. However, utilization of these sample cohorts is difficult due to limited/unavailable clinical data, ambiguous analysis pipelines, and discrepant gene annotations. Here, our first goal was to establish a breast cancer proteomic resource database by combining samples from multiple large independent studies. Then, we aimed to utilize this resource to validate and rank prognostic protein biomarkers in breast cancer.

## Material and methods

### Construction of the integrated protein database

**Figure 1:**
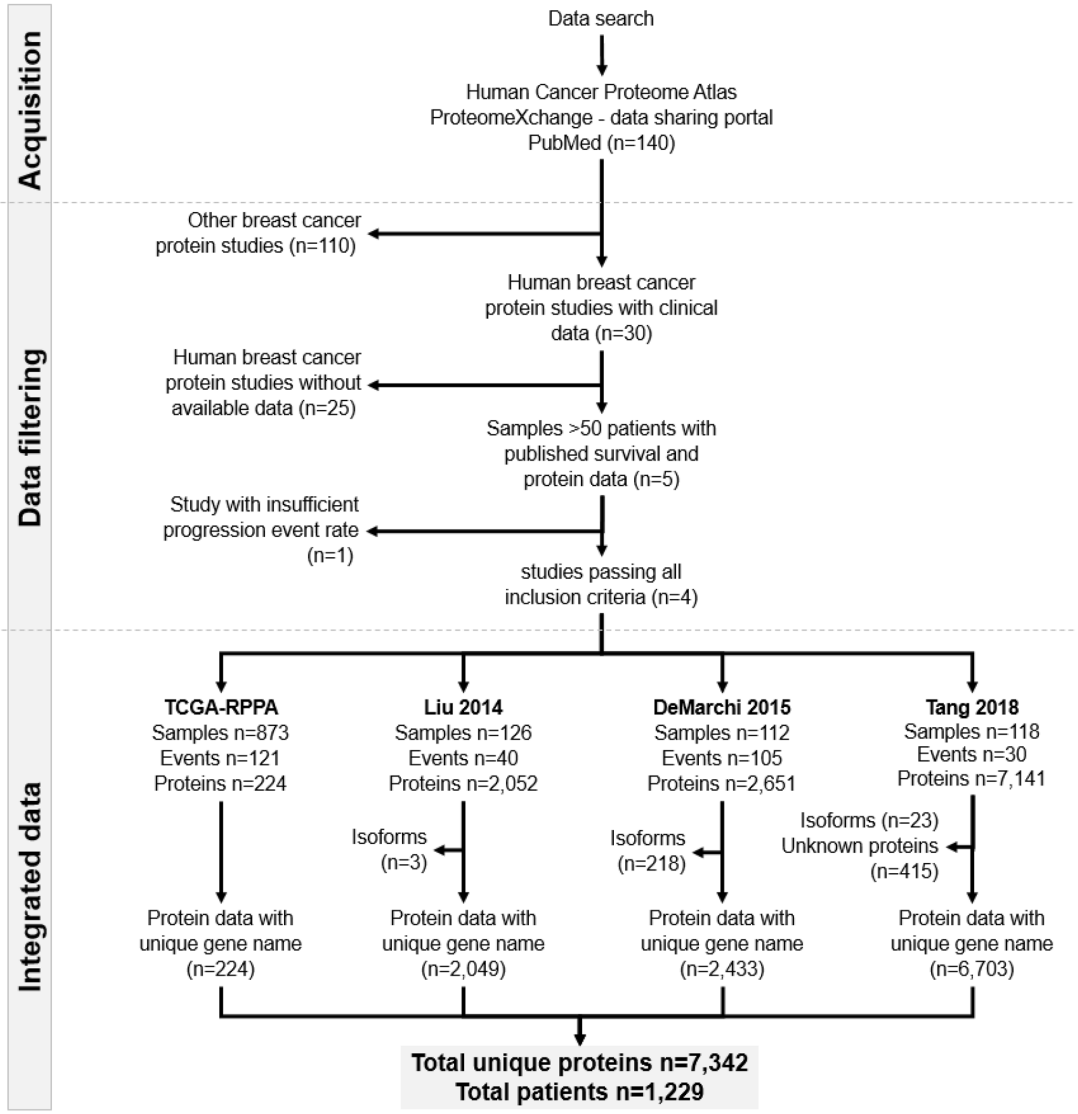
Data acquisition workflow, the number of samples and unique proteins in each included dataset.

We searched for publications and datasets containing proteome and survival data for breast cancer patients in PubMed, The Cancer Proteome Atlas (TCPA)^12^ and the ProteomeXchange Consortium^21^ portals. The search terms “human”, “breast”, and “cancer” were used to identify eligible datasets. Only studies with available protein expression data generated by either mass spectrometry or RPPA, clinical survival information, and at least 50 cancer patients with at least 30 events (either death or relapse) met our inclusion criteria. Four protein datasets met these conditions^12,22–24^. Due to the use of different platforms and analysis methods, it was not possible to merge the datasets into a single unified dataset. Therefore, each dataset was processed separately. In the analyses, the author-reported normalized expression data were used. Figure 1 summarizes the pipeline of data filtering and Supplemental Table 1 summarizes the methods used in the original studies.

**Table 1:** Overview of breast cancer proteomic studies.

Reference	ProteomeXchange /CPTAC ID	Method used	Survival	Sample n	Protein n	Reason for exclusion	Eligible
Tang et al. (2018)	PXD005692	LC–MS/MS	Available	65	7141	–	Yes
Terunuma et al. (2014)	NA	GC–MS, LC–MS	Available	67	NA	No protein data	No
Mertins et al. (2016)	S039 (CPTAC)	LC–MS/MS	Available	105	15,369	Only 13 events	No
Huang et al. (2017)	S032 (CPTAC)	LC–MS/MS	Not available	24	12,794	No survival data	No
Waldemarson et al. (2016)	PXD000944	2D-DIGE, LC–MS/MS	Available	38	14,000	Only 38 samples	No
Cifani et al. (2015)	PXD000691	2D-DIGE, LC–MS/MS	Available	38	3681	Only 38 samples	No
Liu et al. (2014)a	PXD000260	nLC-MS/MS	Available	126	2052	–	Yes
Liu et al. (2014)b	PXD000260	nLC-MS/MS	Available	126	2052	–	Yes
TCGA (2012)	NA	RPPA	Available	348	171	–	Yes
Bouchal et al. (2015)	PXD000029	iTRAQ-2DLC-MS/MS	Not available	96	4405	No survival data	No
Sjöström et al. (2015)	PXD001685	LC–MS/MS; LC-SRM	Not available	80	778	No survival data	No
De Marchi et al. (2015)	PXD000485	LC–MS/MS	Available	112	3109	–	Yes
De Marchi et al. (2016)	PXD002381	LC–MS/MS	Not available	38	3404	No survival data	No
De Marchi et al. (2016)	PXD002381	LC–MS/MS	Not available	38	4	No survival data	No
Pozniak et al. (2016)	PXD000815	LC–MS/MS	Not available	44	10,124	No survival data	No
Pedersen et al. (2017)	PXD005544	TMT-HILIC; LC–MS/MS	Not available	34	4163	No survival data	No
Zagorec et al. (2018)	PXD008012	Ti(IV)-IMAC; LC–MS/MS	Not available	34	2643	No survival data	No
Tyanova et al. (2016)	PXD002619	LC–MS/MS	Not available	40	10,135	No survival data	No
Jiang et al. (2015)	PXD002208	LC–MS/MS	Not available	53	115	No survival data	No
Haukaas et al. (2015)	NA	RPPA	Not available	191	150	No survival data	No
Ternette et al. (2018)	PXD009738	nUPLC‐MS/MS	Not available	11	6275	No survival data	No
Chen et al. (2018)	PXD007217	LC–MS/MS	Not available	10	388	No survival data	No
Naba et al. (2017)	PXD005554	LC–MS/MS	Not available	4	1000	No survival data	No
Gajbhiye et al. (2017)	PXD006441	iTRAQ-SCX; LC–MS/MS	Not available	76	365	No survival data	No
Chen et al. (2018)	PXD007572	LC–MS/MS	Not available	56	556	No survival data	No
Chen et al. (2017)	PXD005214	LC–MS/MS	Not available	36	2413	No survival data	No
Lobo et al. (2017)	PXD003106	LC/MS–MS	Not available	40	4175	No survival data	No
Braakman et al. (2017)	PXD003632	nLC/MS–MS	Not available	38	2995	No survival data	No
Muraoka et al. (2013)	PXD000066	nLC–MS/MS	Not available	18	7092	No survival data	No
Jordan et al. (2016)	PXD003322	SPS-based MS3	Not available	3	6349	No survival data	No

### Protein annotation

In each dataset, the protein annotation generated by the authors was the starting point and duplicated and non-annotated proteins were removed. In addition, UniProt IDs were used to identify gene symbols corresponding to the same genes. The final integrated table of all annotated proteins in the database, including the gene symbol, UniProt ID and TCPA antibody list, is provided as Supplemental Table 2.

**Table 2:** Detailed clinical features of the four protein datasets eligible for this analysis.

Dataset (Reference)	Platform (Company)	Technology	Sample size	Median follow-up (OS, months)	Progression events (OS)	Median follow-up (RFS, months)	Progression events (RFS)	ESR1 + (*)	PGR + (*)	HER2 + (*)	Stage (1/2/3/4)	Grade (1/2/3)	Lymph-node positive	Age	Radiation therapy	Hormone therapy	Chemo-therapy
Cancer Genome Atlas, Li, Lu et al. (2013)	2470 Arrayer (Quanterix)	RPPA	873	27.6	121	25.3	64	627	532	133	128/505/207/18	–	452	58.2 ± 13.3	53	422	488
Liu, Stingl et al. (2014)	LTQ-Orbitrap-XL MS system (ThermoElectron)	LC–MS/MS	126	96.5	40	85.5	50	0	0	0	–	2/16/87	0	53.9 ± 13.8	–	0	0
De Marchi, Liu et al. (2016)	LTQ-Orbitrap-XL MS system (ThermoElectron)	LC–MS/MS	112	–	–	9.6	105	112	–	–	–	–	104	61.1 ± 11.2	–	112	–
Tang, Zhou et al. (2018)	LTQ MS system (Thermo Fisher Scientific)	LC–MS/MS	118	50.0	30	–	–	32	–	–	6/46/13/0	8/19/28	27	54.5 ± 15.7	–	–	–

OS: overall survival, RFS: relapse-free survival.

*ER, PGR, HER2 receptor status was identified using both gene expression and immunohistochemistry data in each cohort.

### Validation of proteome-based protein level determination

To determine how effective recent proteomic technologies could be used in clinical diagnostics in assessing the actual protein levels, we compared proteome-based results to classification (positive/negative) acquired by conventional immunohistochemistry methods. The patient-level data necessary for this analysis was available in multiple data sets for genes with therapeutic importance, including ESR1, PGR, HER2, and MKI67. All validation analyses were performed in each of the four cohorts separately. In the case of MKI67, we also compared the expression between normal and tumor tissue, as this was available in one dataset.

### Correlation between protein biomarker candidates and survival

We performed a PubMed search to identify biomarker candidates related to survival using the search terms “breast cancer”, “protein”, “cohort”, “marker”, and “survival” published up to 2019. Publications describing cell lines, other tumor types, those not investigating a tumor tissue, and studies with fewer than 100 patients were excluded. After these restrictions, 53 publications remained. In addition, we examined ten additional publications describing breast cancer guidelines. In all 63 publications, a total of 91 proteins were linked to breast cancer outcome, 57 of which were present in our database. The identification of the proteins was based on their Uniprot IDs. The list includes FDA-approved biomarkers, growth factor receptors, immune receptor ligands, basal and adhesion markers (cytokeratins, cadherins, and claudins), stem cell markers, and apoptotic markers (Supplemental Table 3). We analyzed all together 63 protein biomarkers used in breast cancer diagnostics for their prognostic power. The validation of the markers was performed separately in each dataset using overall survival and relapse-free survival time.

**Table 3:** Protein markers with validated prognostic value in breast cancer when assessing the correlation between expression level and overall survival (**A**) and relapse-free survival (**B**). *Bold: significant at p* < *0.05.*

(A) Overall Survival			TCGA-RPPA				Liu 2014				Tang 2018			
Protein marker	Symbol	Uniprot ID	n	HR	95% CI	*p* value	n	HR	95% CI	*p* value	n	HR	95% CI	*p* value
Estrogen receptor	ESR1	P03372	733	0.82	0.55–1.21	0.31	–	–	–	–	65	1.53	0.72–3.26	0.27
Progesterone receptor	PGR	P06401	873	1.27	0.85–1.89	0.24	–	–	–	–	**65**	**2.23**	**1.01–4.94**	**0.042**
Human epidermal growth factor receptor 2	HER2	P04626	836	1.32	0.9–1.95	0.16	–	–	–	–	65	1.37	0.64–2.92	0.41
	HER2_pY1248		**871**	**1.63**	**1.13–2.36**	**0.0079**								
Androgen receptor	AR	P10275	870	1.37	0.88–2.14	0.16	–	–	–	–	**65**	**0.29**	**0.1–0.83**	**0.014**
Apoptosis Regulator, BCL2	BCL2	P10415	**869**	**0.56**	**0.39–0.81**	**0.0017**	–	–	–	–	–	–	–	–
Basal markers, Cytokeratin-8	KRT8	P05787	–	–	–	–	125	1.86	0.99–3.49	0.051	**65**	**2.16**	**1.03–4.55**	**0.038**
Basal markers, Cytokeratin-18	KRT18	P05783	–	–	–	–	**126**	**0.35**	**0.14–0.88**	**0.02**	**65**	**2.35**	**1.11–5.00**	**0.022**
Basal markers, Cytokeratin-5	KRT5	P13647	–	–	–	–	126	0.54	0.29–1.01	0.05	**65**	**0.41**	**0.19–0.85**	**0.014**
Basal markers, Cytokeratin-6A	KRT6A	P02538	–	–	–	–	121	0.63	0.3–1.33	0.22	**65**	**2.17**	**1.02–4.61**	**0.039**
Basal markers, Cytokeratin-6B	KRT6B	P04259	–	–	–	–	**115**	**0.46**	**0.23–0.9**	**0.019**	65	1.89	0.91–3.9	0.081
Basal markers, Cytokeratin-17	KRT17	Q04695	–	–	–	–	**126**	**0.49**	**0.26–0.92**	**0.022**	65	1.59	0.61–4.16	0.34
Adhesion marker, E-Cadherin	CDH1	P12830	**668**	**1.76**	**1.07–2.89**	**0.024**	**125**	**0.21**	**0.08–0.6**	**0.0013**	65	0.58	0.28–1.2	0.14
Adhesion markers, Claudin-3	CLDN3	O15551	–	–	–	–	**119**	**0.48**	**0.26–0.91**	**0.021**	–	–	–	–
Transcription factor, Y-box-binding protein 1	YBX1	P67809	872	0.73	0.5–1.07	0.11	–	–	–	–	**65**	**2.07**	**0.99–4.31**	**0.047**
	YBX1_pS102		**873**	**1.48**	**1.0–2.17**	**0.046**	–	–	–	–	–	–	–	–
Invasion marker, Stromelysin-3	MMP11	P24347	–	–	–	–	–	–	–	–	**65**	**2.09**	**1.0–4.35**	**0.044**
N-myc downstream-regulated gene 1 protein	NDRG1	Q92597	–	–	–	–	126	0.66	0.34–1.28	0.216	**65**	**2.24**	**1.07–4.72**	**0.0288**
Catenin beta-1	CTNNB1	P35222	873	1.38	0.84–2.29	0.2031	**126**	**0.27**	**0.12–0.59**	**4E–04**	65	1.73	0.74–4.07	0.2009
Apolipoprotein D	APOD	P05090	–	–	–	–	**126**	**0.56**	**0.29–1.08**	**0.081**	**65**	**0.35**	**0.12–1.0**	**0.0411**
Poly [ADP-ribose] polymerase 1	PARP1	P09874	873	1.55	0.77–3.09	0.2134	126	0.79	0.93–3.48	0.079	**65**	**2.44**	**1.18–5.05**	**0.0131**
Scavenger receptor cysteine-rich type 1 protein M130	CD163	Q86VB7	–	–	–	–	126	0.74	0.35–1.53	0.412	**65**	**2.43**	**1.17–5.06**	**0.0138**
Fascin	FSCN1	Q16658	–	–	–	–	**126**	**0.52**	**0.28–0.98**	**0.040**	**65**	**2.52**	**1.2–5.26**	**0.0111**
Asporin	ASPN	Q9BXN1	–	–	–	–	–	–	–	–	**65**	**2.29**	**1.06–4.94**	**0.0294**
RNA-binding protein 3	RBM3	P98179	–	–	–	–	**126**	**0.42**	**0.18–1.01**	**0.045**	65	2.03	0.97–4.26	0.056
Glioma-associated oncogene	GLI1	P08151	–	–	–	–	–	–	–	–	**65**	**0.43**	**0.18–1**	**0.0427**

(B) Relapse-free survival			TCGA-RPPA				Liu 2014				De Marchi 2015			
Protein marker	Symbol	Uniprot ID	n	HR	95% CI	*p* value	n	HR	95% CI	*p* value	n	HR	95% CI	*p* value
Estrogen receptor	ESR1	P03372	623	0.64	0.36–1.14	0.13	–	–	–	–	**112**	**0.3**	**0.19–0.49**	**1.9e − 07**
Progesterone receptor	PGR	P06401	**750**	**0.42**	**0.26–0.69**	**0.0004**	–	–	–	–	**112**	**0.61**	**0.41–0.92**	**0.018**
Human epidermal growth factor receptor 2	HER2	P04626	719	1.19	0.73–1.96	0.48	–	–	–	–	112	0.75	0.51–1.11	0.15
	HER2_pY1248		748	0.68	0.39–1.21	0.19								
Apoptosis Regulator, BCL2	BCL2	P10415	**746**	**0.51**	**0.31–0.84**	**0.0071**	–	–	–	–	**112**	**0.4**	**0.27–0.61**	**9.5e − 06**
Basal markers, Cytokeratin-18	KRT18	P05783	–	–	–	–	**124**	**0.39**	**0.17–0.86**	**0.016**	–	–	–	–
Basal markers, Cytokeratin-5	KRT5	P13647	–	–	–	–	**124**	**0.49**	**0.28–0.85**	**0.01**	–	–	–	–
Basal markers, Cytokeratin-6B	KRT6B	P04259	–	–	–	–	**113**	**0.43**	**0.23–0.77**	**0.004**	–	–	–	–
Basal markers, Cytokeratin-17	KRT17	Q04695	–	–	–	–	**124**	**0.51**	**0.29–0.88**	**0.014**	–	–	–	–
Adhesion marker, E-Cadherin	CDH1	P12830	578	1.83	0.93–3.58	0.075	**123**	**0.35**	**0.16–0.78**	**0.007**	**112**	**0.61**	**0.39–0.95**	**0.026**
Adhesion markers, Claudin-7	CLDN7	O95471	**715**	**1.67**	**1–2.79**	**0.048**	–	–	–	–	112	0.72	0.49–1.06	0.098
Apoptotic marker, Tumorsupressor p53	TP53	P04637	**727**	**1.84**	**1.12–3.02**	**0.014**	–	–	–	–	–	–	–	–
Bcl-2-associated athanogene 1	BAG1	Q99933	–	–	–	–	–	–	–	–	**112**	**0.58**	**0.39–0.86**	**0.0061**
Carcinoembryonic antigen-related cell adhesion molecule 5	CEACAM5	P06731	–	–	–	–	–	–	–	–	**112**	**0.66**	**0.43–1.00**	**0.049**
N-myc downstream-regulated gene 1 protein	NDRG1	Q92597	–	–	–	–	124	0.58	0.33–1.03	0.059	**112**	**0.56**	**0.37–0.87**	**0.0084**
Large neutral amino acids transporter small subunit 1	SLC7A5	Q01650	–	–	–	–	–	–	–	–	**112**	**1.5**	**1.01–2.22**	**0.0455**
Catenin beta-1	CTNNB1	P35222	750	0.73	0.71–1.3	0.2823	**124**	**0.36**	**0.19–0.7**	**0.002**	**112**	**0.56**	**0.36–0.85**	**0.0061**
Apolipoprotein D	APOD	P05090	–	–	–	–	124	0.64	0.35–1.15	0.133	**112**	**0.59**	**0.38–0.91**	**0.0161**
Poly [ADP-ribose] polymerase 1	PARP1	P09874	750	0.46	0.17–1.3	0.1341	124	1.49	0.83–2.66	0.176	**112**	**0.65**	**0.44–0.97**	**0.0349**
Carcinoembryonic antigen-related cell adhesion molecule 6	CEACAM6	P40199	–	–	–	–	–	–	–	–	**112**	**0.56**	**0.38–0.84**	**0.0044**
Ras-related protein Rab-27B	RAB27B	O00194	–	–	–	–	–	–	–	–	**112**	**0.59**	**0.37–0.92**	**0.0183**
RNA-binding protein 3	RBM3	P98179	–	–	–	–	**124**	**0.40**	**0.19–0.86**	**0.016**	112	0.78	0.51–1.18	0.2466
GATA-binding factor 3	GATA3	P23771	750	0.61	0.37–1.01	0.0544	–	–	–	–	**112**	**0.49**	**0.32–0.74**	**0.0007**

### Statistical analyses

The immunohistochemistry classification was available as positive/negative and we used this classification to divide the samples into two groups. The differential expression between these groups was evaluated using the Mann–Whitney test by comparing the variables in each study separately. In a second analysis, Receiver operating characteristics (ROC) were computed to measure sensitivity and specificity and to validate discriminative power. ROC was also utilized to determine the optimal cutoff values to define cohorts based on the expression of the investigated proteins. Spearman rank correlation coefficients were calculated to assess the correlation of continuous variables. To measure the association between protein expression and survival length, the patients were grouped into high and low expression groups based on the expression of the selected protein. Then, the two groups were compared by Cox proportional hazards regression, and hazard ratios (HRs), 95% confidence intervals (CIs) and log-rank *p* values were calculated. Finally, for a selected set of markers, Kaplan–Meier plots were generated to display the different survival characteristics of the two cohorts^25^. For cutoff values, each potential threshold was analyzed between the lower and upper quartiles, and the false discovery rate (FDR) was computed to correct for multiple hypothesis testing. The results were accepted as significant when *p* < 0.05 and FDR < 0.2.

### Survival analysis web tool

We previously created an online analysis platform utilizing transcriptome-level mRNA expression^26^ and miRNA expression^27^ data together with clinical, follow-up, and pathological data to assess the correlation between gene expression and survival in breast cancer. Here, we have established a new subsystem of this analysis platform. The complete proteomic database is now integrated into this system, and new biomarker candidates, as well as each biomarker assessed here, can be rapidly evaluated using the registration-free analysis site. In the tool, selection of the proteins can be performed using the gene symbol, the UniProt ID or the RPPA antibody name (https://kmplot.com/analysis/).

## Results

### Integrated breast cancer protein database

**Figure 2:**
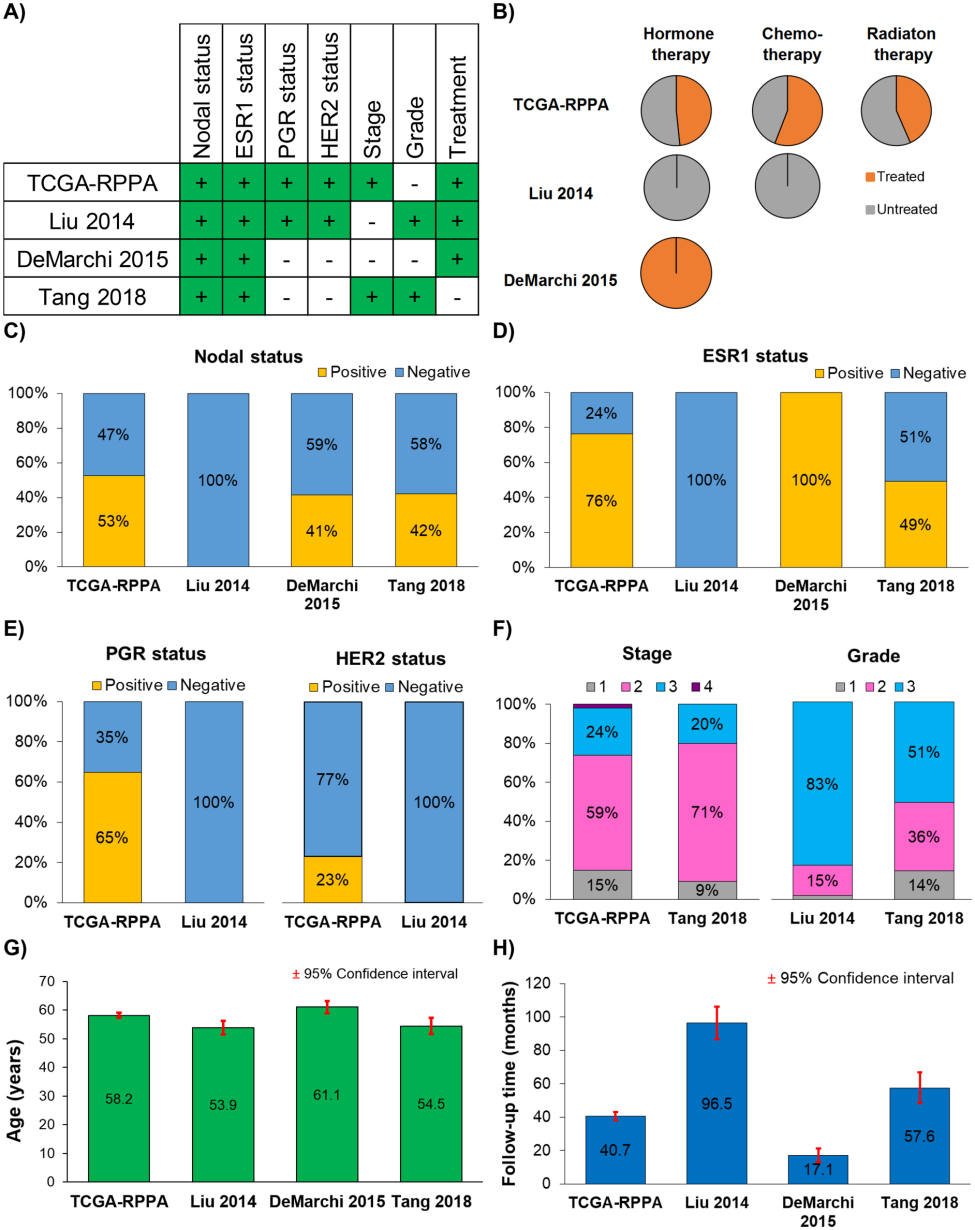
Clinical characteristics of the breast cancer patients used in this study. (**A**) Availability of clinical data in the included cohorts; (**B**) the proportion of patients treated with radiation, hormones or chemotherapy. (**C**) Percentage of patients by nodal status in each dataset; (**D**)**, **(**E**) the proportion of patients by receptor status for ESR1, PGR and HER2 in each dataset; (**F**) the distribution of stage and grade; (**G**) the mean age of patients; and (**H**) the mean follow-up time in each dataset.

Altogether, 140 datasets were identified, of which 30 studies had at least some clinical information for the included patients. We listed all these datasets in Table 1. After exclusion of those without survival data and other ineligible studies, four independent projects remained. These four datasets comprise 1229 specimens and 7342 unique proteins. The entire set of patients included 1064 overall survival (OS) and 998 relapse-free survival (RFS) records. Two datasets had either only overall^24^ or relapse-free survival data^23^. Median OS and RFS times varied between 27.6 and 96.5 months and 9.6–85.5 months, respectively. The mean age of the patients was 57.7 ± 13.6 years. In line with previous expectations^28^, estrogen receptor-positive (ESR1 +) patients represented approximately 67% of all samples, and almost half of the patients had nodal involvement (46%). Of note, the Liu 2014 dataset included triple negative breast cancer (TNBC)^22^, lymph node negative and treatment naive patients only. In the other studies, hormone therapy, primarily tamoxifen, was applied (59%). Table 2 contains detailed clinical parameters for each included dataset used, and Fig. 2 shows selected clinical characteristics for these datasets.

**Figure 3:**
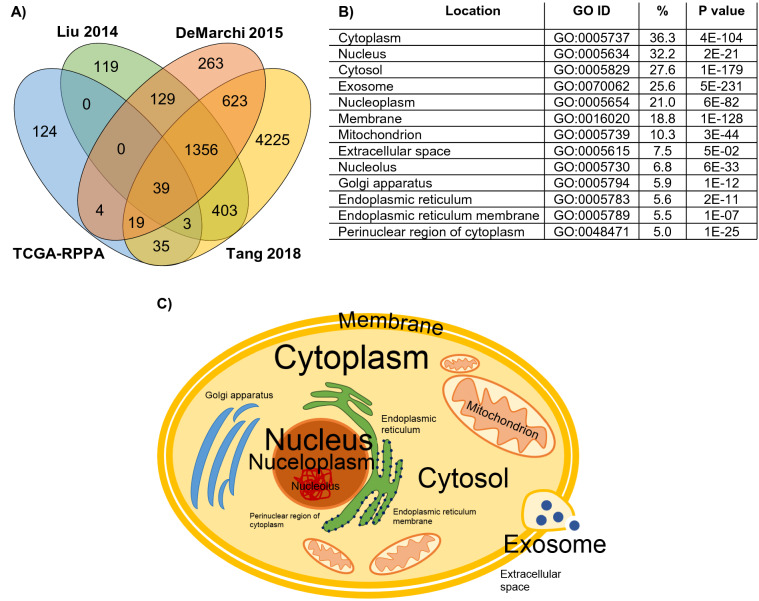
Proteins measured in multiple studies and their cellular localizations. (**A**) Number of proteins represented in one, two, three, or four datasets, (**B**) proportion of proteins present in various cellular components, and (**C**) graphical representation of cellular origin of the analyzed proteins, where font size is relative to the proportion of proteins from that compartment.

The dataset generated using RPPA contains most of the patients (n = 873) but least of the proteins (n = 224). The other three datasets have combined > 7000 protein records measured by LC–MS/MS technology. Figure 3A shows the proportions of detected proteins in each dataset combination. Only 39 proteins were measured in all datasets, while 1356 overlapping proteins were evaluated in the three LC–MS/MS studies. A total of 4731 proteins were detected in only one study, and most of them came from the Tang 2018 cohort (n = 4225)^24^. When mapping the measured proteins to cellular locations, the majority of proteins originated from the cytoplasm (36.3%), nucleus (32.2%) and cytosol (27.6%) (Fig. 3B,C). Supplemental Table 2 includes all proteins.

### Evaluation of routine diagnostic biomarkers

**Figure 4:**
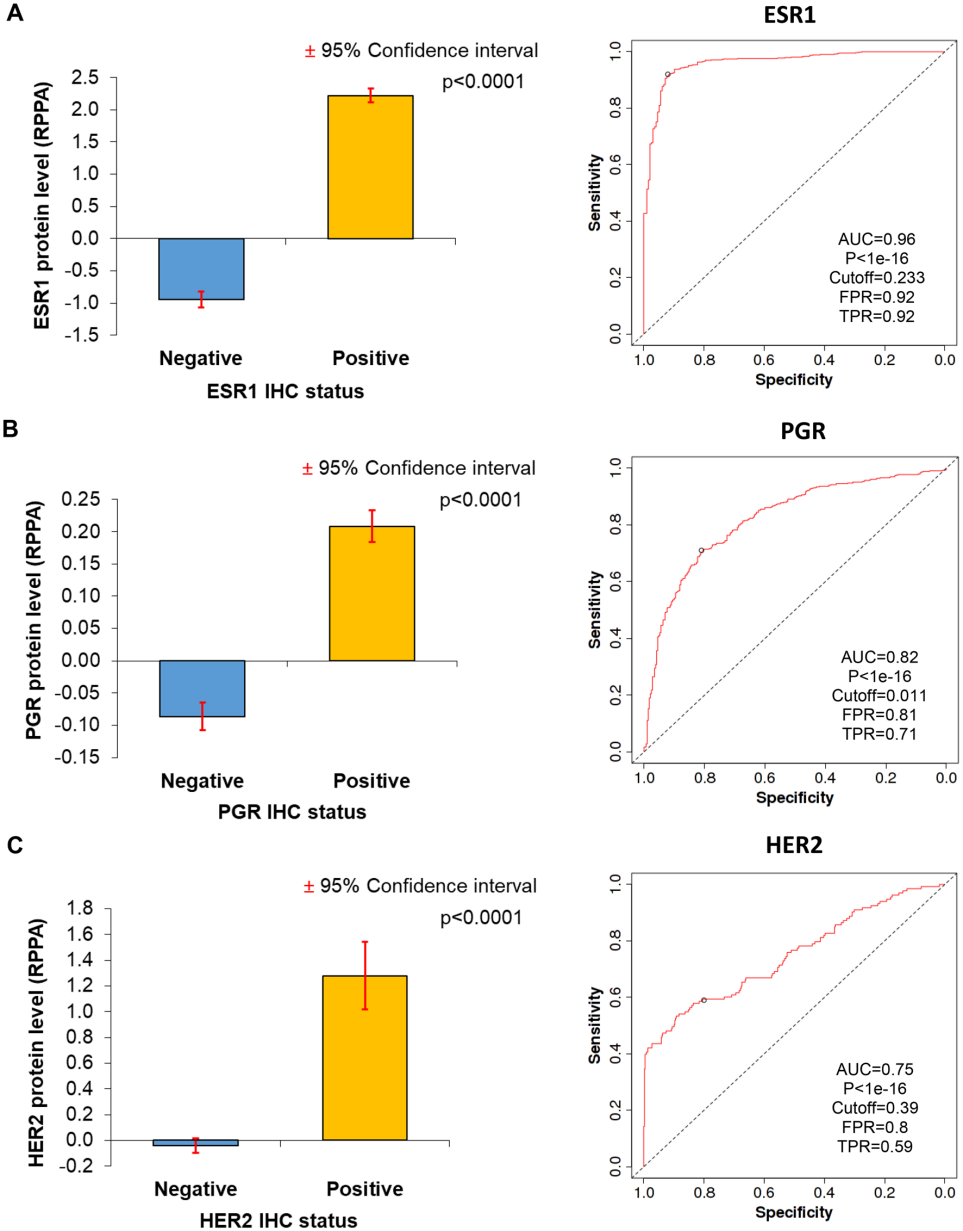
Validation of proteome-based molecular biomarker determination by comparing the results achieved by IHC-based receptor status determination (positive or negative) to data generated by RPPA. Means plots and ROC curves for ESR1 (**A**), PGR (**B**), and HER2 (**C**) protein expression results determined by RPPA show a significant correlation with IHC results. *AUC* area under the curve, *FPR* false positive rate, *TPR* true positive rate.

**Figure 5:**
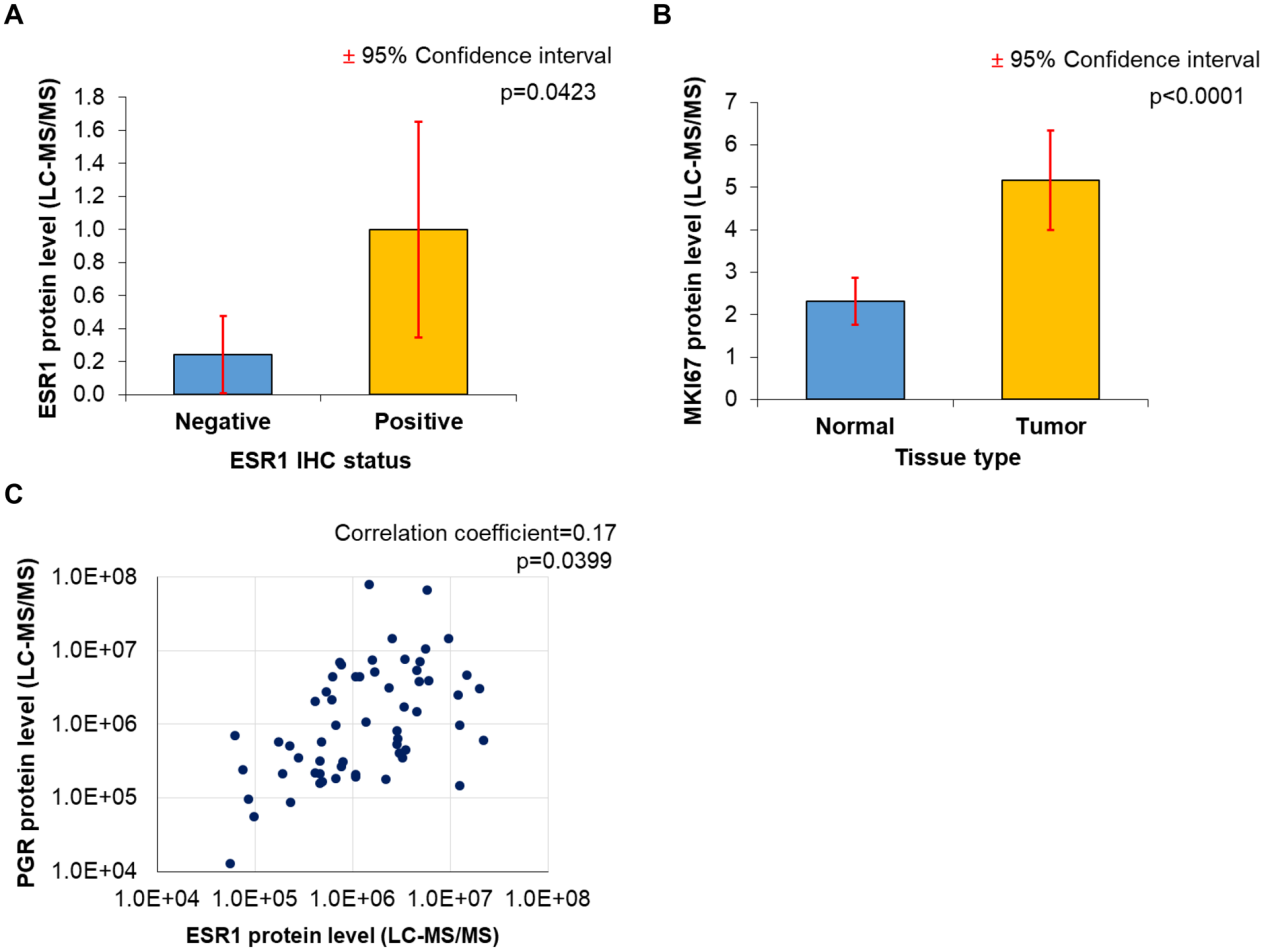
The correlation between ESR1 status by IHC and ESR1 protein expression levels measured by LC–MS/MS (**A**). MKI67 levels measured by LC–MS/MS showed higher expression in tumors than in normal samples in the Tang 2018 dataset (n = 53) (**B**)**.** Correlation between ESR1 and PGR protein expression levels in LC–MS/MS data (**C**).

Immunohistochemistry results were available as positive or negative, and we compared the expression of the selected protein (e.g. ESR1) between the positive and the negative groups. When ESR1, PGR, and HER2 protein expression levels determined by RPPA were compared to IHC-based receptor status, results revealed that protein expression and receptor status were highly significantly correlated with one another (*p* < 0.0001) (see means-plots and ROC-plots in Fig. 4A–C). When running ROC analysis using RPPA-based continuous HER2 levels, the proteomic measurements delivered a substantial area under the ROC curve (AUC) of 0.74 (*p* = 1.9e−20). ESR1 protein expression determined by LC–MS/MS also delivered a correlation to IHC results (*p* = 0.0423) (Fig. 5A). The AUC value for ESR1 levels determined by LC–MS/MS was 0.61 (*p* = 0.03). Thus, the AUC values for LC–MS/MS were much lower than the RPPA-based AUC values showing the higher dynamic range of RPPA. The Tang et al. dataset included paired normal and tumor samples for 53 patients^24^. When comparing the expression of the proliferation marker MKI67 between the normal and cancer samples, the tumor samples had significantly higher expression (fold change = 2.22, *p* = 0.0001) (Fig. 5B).

Finally, we also assessed the correlation between ESR1 and the ESR1-regulated gene PGR. In this analysis, we uncovered a moderate correlation between ESR1 and PGR protein expression levels, as determined by LC–MS/MS (correlation coefficient = 0.17, *p* = 0.0399, Fig. 5C). Unfortunately, due to the limited availability of simultaneously collected data, it was not possible to analyze all possible clinical scenarios and to model molecular subtype determination based on proteomic datasets.

### Proteins with significant prognostic power

We assessed the link between survival and the expression of 63 proteins and their phosphorylated forms to validate their prognostic relevance in breast cancer (Supplemental Table 3). The expression of 33 of 63 proteins had a significant correlation with patient outcome. Twelve proteins associated with OS only, nine proteins associated with RFS only, and twelve proteins (PGR, CDH1, BCL2, NDRG1, CTNNB1, APOD, PARP1, RBM3 and four cytokeratins: KRT18, KRT5, KRT6B, KRT17) were prognostic for both RFS and OS. Of these, three proteins (KRT18, APOD and CDH1) and four proteins (PGR, CDH1, CTNNB1, and BCL2) were confirmed to be related to OS and RFS, respectively, in at least two independent datasets. The results of the survival analysis for each of these proteins in terms of OS and RFS are displayed in Table 3A and 3B, respectively.

**Figure 6:**
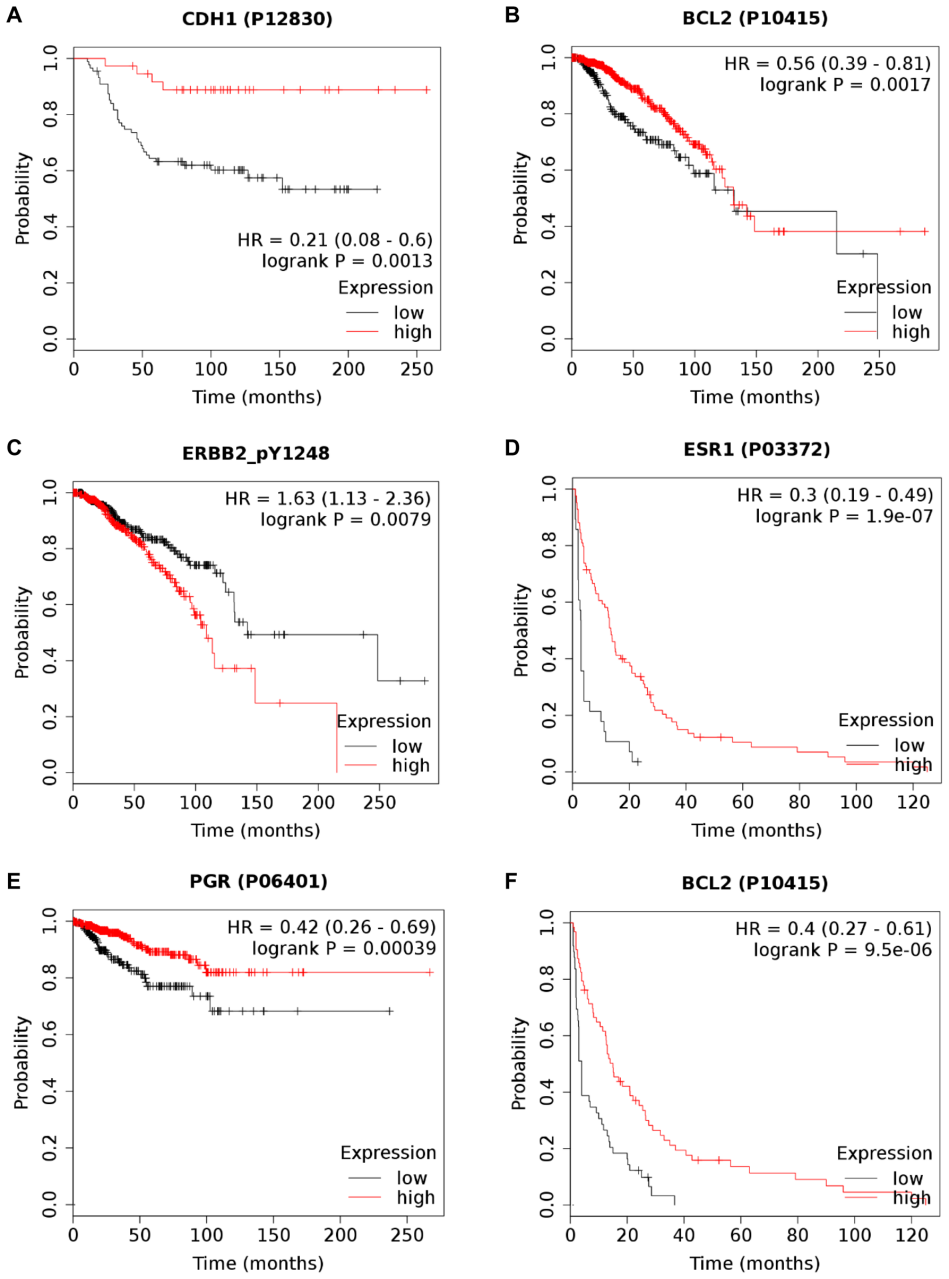
Survival outcome differences in patients with different expression levels of protein biomarkers. Kaplan–Meier plots of overall survival by CDH1 (E-cadherin) (**A**), apoptosis regulator BCL2 (**B**), and tyrosine 1248-phosphorylated HER2 (**C**). Kaplan–Meier plots of relapse-free survival for estrogen receptor 1 (**D**), progesterone receptor (**E**) and BCL2 (**F**) in breast cancer patients. Note the different survival characteristics of the different datasets.

A better overall survival outcome was associated with higher expression of E-cadherin (HR = 0.21, 95%CI = 0.08 − 0.6, *p* = 0.0013) and the apoptosis regulator protein BCL2 (HR = 0.6, 95%CI = 0.39 − 0.81, *p* = 0.0017). Higher BCL2 was also strongly related to longer relapse-free survival (HR = 0.4, 95%CI = 0.27 − 0.61, *p* = 9.5e − 06). While we also validated the prognostic value of the expression level of tyrosine 1248-phosphorylated HER-2 (HER2_pY1248) (HR = 1.63, 95%CI = 1.13 − 2.36, *p* = 0.0079) using RPPA data, the expression level of nonphosphorylated HER-2 did not have a significant correlation with survival in any of the included datasets. Both estrogen receptor and progesterone receptor were linked to improved relapse-free survival (HR = 0.3, 95%CI = 0.19 − 0.49, *p* = 1.9e − 07 and HR = 0.4, 95%CI = 0.26 − 0.69, *p* = 0.0004, respectively). Kaplan–Meier curves for these proteins are shown in Fig. 6A–F.

## Discussion

A major advance of proteomic technologies lies in their ability to simultaneously measure multiple biomarkers from a single clinical specimen. Here, we collected four independent breast cancer proteomic cohorts and validated established and new biomarker candidates.

Despite the quantitative and multiplexing limitations of immunohistochemical analysis, in clinical practice, it is still the gold standard. We compared the efficiency of various proteomic techniques to determine routinely measured breast cancer biomarkers, including ESR1, PGR, HER2, and MKI67. In this analysis, both the RPPA and LC–MS/MS method results were highly correlated with IHC results and thus can be utilized to determine receptor status in breast cancer patients. Unfortunately, we did not have all markers for the same patients, and the results achieved for individual genes can only suggest that proteomic technologies will also be capable of performing molecular stratification in the future, enabling the discrimination of breast cancer subtypes.

Estrogen receptor is a pioneer cancer biomarker, and classifying breast tumors based on hormone receptor status has been utilized in routine clinical practice for over four decades^29^. ESR1 positivity and PGR positivity are associated with better survival outcomes than negative ESR1/PGR status. In addition to clinicopathological prognostication, the main medical application of these receptors is selecting patients for endocrine therapy^30^.

MKI67 is a protein not expressed in G0 phase, and thus, it is a perfect marker for determining the proportion of dividing cells^31^. MKI67 expression is correlated with outcome, and high MKI67 expression is associated with poor prognosis, which has been validated in a meta-analysis involving over 64 thousand breast cancer patients^32^. Immunohistochemical staining of MKI67 alone can also pinpoint low-risk breast cancers with the same reliability as genomic markers^33^.

Evaluation of HER2 (ERBB2, neu) status has also been routinely used in breast cancer molecular diagnostics since the end of the 1990s. Analysis of large cohorts of patients found that HER2 overexpression is associated with unfavorable prognosis and poor response to chemotherapy^34^. The clinical introduction of anti-HER2 therapies (i.e., trastuzumab, pertuzumab) in combination with chemotherapy in patients who have HER2-positive cancer results in exceptional survival advantages. As a result, HER2-positive patients have a better outlook than HER2-negative patients^35^. Today, tumors with even 1% positivity are eligible for anti-HER2 therapy^36^.

Triple-negative breast cancer (TNBC) is diagnosed in cases where tumors are negative for ESR1, PGR, and HER2. In these breast tumors, the immunohistochemical measurement of basal markers (cytokeratin 5/6, EGFR), claudins (CLD3/4/7), cadherins (CDH1, CDH3), stem cell markers (CD44/CD24, ALDH1), apoptosis markers (BCL2, TP53), a transcription marker (YB-1) and urokinase-type plasminogen activator (uPA)/plasminogen activator inhibitor-1 (PAI-1) have also been suggested for advanced stratification^19,20,37,38^.

We assessed the prognostic power of a selected set of proteins, including ESR1, PGR, HER2, cytokeratins, claudins, E-cadherin^39^ and EGFR, in the datasets included in the present study. Overall, we uncovered that 33 proteins had a significant correlation with prognosis. In the case of FDA-approved protein biomarkers, the expression of estrogen and progesterone receptors is correlated with favorable relapse-free survival. High expression levels of phosphorylated HER2 protein measured by RPPA were linked with worse overall survival than low expression levels; these findings are in line with the previous study by Hayashi et al. on the same protein^40^.

High expression of the antiapoptotic Bcl-2 and the adhesion marker E-cadherin was related to longer relapse-free survival than low expression in at least two independent datasets. Bcl-2 overexpression was revealed in other cancers and was linked to cancer initiation and progression, and higher expression positively correlated with favorable patient outcomes in hormone receptor-positive breast tumors^41,42^. Loss of E-cadherin expression is frequently represented in invasive lobular breast carcinoma, which is three times more likely to metastasize^43^.

Interestingly, some of the genes, including PGR and E-cadherin, display inverse correlations with survival when assessing the link to survival in different patient cohorts. Here, we have to mention some limitations of our analysis that might lie behind these discrepancies. A major constraint is that only 20% of the proteins were determined in at least three platforms. This means that the evaluation of further databases will be needed to perform a comprehensive validation of all potential biomarker candidates. Another shortcoming of the investigated datasets is the rather low proportion of events (in the case of the TCGA dataset)^12^ and the short follow-up time (DeMarchi dataset^23^). A future large-scale proteomic database with long follow-up and uniform protein level determination using a single method could provide more reliable data for a similar analysis.

In summary, we successfully integrated four distinct breast cancer proteomic datasets containing tumor and normal samples. A significant correlation was observed between marker levels detected by proteomic technologies and those detected by immunohistochemistry results. We validated prognostic and predictive breast cancer biomarkers and compared the efficiency of different proteome analysis techniques. The entire database is integrated into our online tool, providing an opportunity to validate our findings and to identify and rank new survival-associated biomarker candidates using multiple independent cohorts of breast cancer.

## Supplementary Information

Supplementary Information 1.

Supplementary Information 2.

Supplementary Information 3.

Supplementary Information 4.

## References

[1] Bray F (2018). Global cancer statistics 2018: GLOBOCAN estimates of incidence and mortality worldwide for 36 cancers in 185 countries. CA. Cancer J. Clin..

[2] Győrffy B (2015). Multigene prognostic tests in breast cancer: past, present, future. Breast Cancer Res..

[3] Berggård T, Linse S, James P (2007). Methods for the detection and analysis of protein–protein interactions. Proteomics.

[4] Solier C, Langen H (2014). Antibody-based proteomics and biomarker research—current status and limitations. Proteomics.

[5] Camp RL, Neumeister V, Rimm DL (2008). A decade of tissue microarrays: progress in the discovery and validation of cancer biomarkers. J. Clin. Oncol..

[6] Boellner S, Becker K-F (2015). Reverse phase protein arrays—quantitative assessment of multiple biomarkers in biopsies for clinical use. Microarrays.

[7] Malinowsky K, Wolff C, Schott C, Becker K-F, Malek A, Tchernitsa O (2013). Characterization of signalling pathways by reverse phase protein arrays. Ovarian Cancer.

[8] Chung L (2014). Novel serum protein biomarker panel revealed by mass spectrometry and its prognostic value in breast cancer. Breast Cancer Res..

[9] Domon B, Aebersold R (2010). Options and considerations when selecting a quantitative proteomics strategy. Nat. Biotechnol..

[10] Rodríguez-Suárez E, Whetton AD (2013). The application of quantification techniques in proteomics for biomedical research: quantification techniques in proteomics. Mass Spectrom. Rev..

[11] *Integrative Proteomics*. (InTech, 2012). 10.5772/2473.

[12] Li J (2013). TCPA: a resource for cancer functional proteomics data. Nat. Methods.

[13] Omenn GS (2018). Progress on identifying and characterizing the human proteome: 2018 metrics from the HUPO human proteome project. J. Proteome Res..

[14] Deutsch EW (2017). The ProteomeXchange consortium in 2017: supporting the cultural change in proteomics public data deposition. Nucleic Acids Res..

[15] Uhlen M (2015). Tissue-based map of the human proteome. Science.

[16] Rivers RC (2014). Linking cancer genome to proteome: NCI’s investment into proteogenomics. Proteomics.

[17] Johnson KS, Conant EF, Soo MS (2021). Molecular subtypes of breast cancer: a review for breast radiologists. J. Breast Imaging.

[18] Vasconcelos I (2016). The St. Gallen surrogate classification for breast cancer subtypes successfully predicts tumor presenting features, nodal involvement, recurrence patterns and disease free survival. The Breast.

[19] Portier BP (2012). From morphologic to molecular: established and emerging molecular diagnostics for breast carcinoma. New Biotechnol..

[20] Mueller C, Haymond A, Davis JB, Williams A, Espina V (2018). Protein biomarkers for subtyping breast cancer and implications for future research. Expert Rev. Proteomics.

[21] Vizcaíno JA (2014). ProteomeXchange provides globally coordinated proteomics data submission and dissemination. Nat. Biotechnol..

[22] Liu, N. Q. *et al.* Comparative proteome analysis revealing an 11-protein signature for aggressive triple-negative breast cancer. *JNCI J. Natl. Cancer Inst.* **106** (2014). 10.1093/jnci/djt376 PMC3952199 24399849

[23] De Marchi T (2016). 4-protein signature predicting tamoxifen treatment outcome in recurrent breast cancer. Mol. Oncol..

[24] Tang W (2018). Integrated proteotranscriptomics of breast cancer reveals globally increased protein-mRNA concordance associated with subtypes and survival. Genome Med..

[25] Kaplan EL, Meier P (1958). Nonparametric estimation from incomplete observations. J. Am. Stat. Assoc..

[26] Györffy B (2010). An online survival analysis tool to rapidly assess the effect of 22,277 genes on breast cancer prognosis using microarray data of 1809 patients. Breast Cancer Res. Treat..

[27] Lánczky A (2016). miRpower: a web-tool to validate survival-associated miRNAs utilizing expression data from 2178 breast cancer patients. Breast Cancer Res. Treat..

[28] Lumachi F, Brunello A, Maruzzo M, Basso U, Basso SMM (2013). Treatment of estrogen receptor-positive breast cancer. Curr. Med. Chem..

[29] Osborne CK, Yochmowitz MG, Knight WA, McGuire WL (1980). The value of estrogen and progesterone receptors in the treatment of breast cancer. Cancer.

[30] Hammond MEH (2010). American Society of Clinical Oncology/College of American Pathologists guideline recommendations for immunohistochemical testing of estrogen and progesterone receptors in breast cancer (unabridged version). Arch. Pathol. Lab. Med..

[31] Miller I (2018). Ki67 is a graded rather than a binary marker of proliferation versus quiescence. Cell Rep..

[32] Petrelli F, Viale G, Cabiddu M, Barni S (2015). Prognostic value of different cut-off levels of Ki-67 in breast cancer: a systematic review and meta-analysis of 64,196 patients. Breast Cancer Res. Treat..

[33] Iwamoto T (2017). Immunohistochemical Ki67 after short-term hormone therapy identifies low-risk breast cancers as reliably as genomic markers. Oncotarget.

[34] Slamon D (1987). Human breast cancer: correlation of relapse and survival with amplification of the HER-2/neu oncogene. Science.

[35] Ross JS (2009). The HER-2 receptor and breast cancer: ten years of targeted anti–HER-2 therapy and personalized medicine. Oncologist.

[36] Wolff AC (2018). Human epidermal growth factor receptor 2 testing in breast cancer: American Society of Clinical Oncology/College of American pathologists clinical practice guideline focused update. J. Clin. Oncol..

[37] Blows FM (2010). Subtyping of Breast Cancer by Immunohistochemistry to investigate a relationship between subtype and short and long term survival: a collaborative analysis of data for 10,159 cases from 12 studies. PLoS Med..

[38] Norum JH, Andersen K, Sørlie T (2014). Lessons learned from the intrinsic subtypes of breast cancer in the quest for precision therapy. Br. J. Surg..

[39] Szasz AM (2011). Identification of a claudin-4 and E-cadherin score to predict prognosis in breast cancer. Cancer Sci..

[40] Hayashi N (2011). Prognostic impact of phosphorylated HER-2 in HER-2 ^+^ primary breast cancer. Oncologist.

[41] Dawson S-J (2010). BCL2 in breast cancer: a favourable prognostic marker across molecular subtypes and independent of adjuvant therapy received. Br. J. Cancer.

[42] Honma N (2015). Differences in clinical importance of Bcl-2 in breast cancer according to hormone receptors status or adjuvant endocrine therapy. BMC Cancer.

[43] Michaut M (2016). Integration of genomic, transcriptomic and proteomic data identifies two biologically distinct subtypes of invasive lobular breast cancer. Sci. Rep..

