# Supplementary material for: Survival analysis in breast cancer using proteomic data from four independent datasets
Source: Sci Rep. 2021 Aug 18;11:16787. doi: 10.1038/s41598-021-96340-5 (PMC8373859; doi:10.1038/s41598-021-96340-5)
Supplement: Supplementary file 1 — Supplementary Information 1. [file 41598_2021_96340_MOESM1_ESM.docx]

**Supplemental Table 1.**

Processing of proteomic data in the four protein datasets eligible for our analysis

| Dataset (Reference) | Platform (Company) | Method | Sample size | Protein number | Protein filtering criteria | Protein expression quantification | Normalized in original study | Reference database |
| --- | --- | --- | --- | --- | --- | --- | --- | --- |
| TCGA-RPPA ^1,2^ | 2470 Arrayer (Quanterix) | RPPA | 873 | 224 | >0.8 QC score | SuperCurve Fitting | Replicates-based normalization | - |
| Liu 2014 ^3^ | LTQ-Orbitrap-XL MS system (ThermoElectron) | LC-MS/MS | 126 | 2049 | Peptides ≥7 amino acid residues (1% FDR). Proteins quantified in ≥60% samples. | Label-free quantification algorithm in MaxQuant | Combat algorithm in R statistical package | UniProtKB human database (version 2011_03) |
| DeMarchi 2015 ^4^ | LTQ-Orbitrap-XL MS system (ThermoElectron) | LC-MS/MS | 112 | 2433 | Peptides ≥7 amino acid residues (1% FDR). | Label-free quantification algorithm in MaxQuant | Combat algorithm in R statistical package | UniProt human database (version 2012_09) |
| Tang 2018 ^5^ | LTQ MS system (Thermo Fisher Scientific) | LC-MS/MS | 118 | 6073 | Peptides: 1% and 5 % FDR. Proteins >10% sample coverage | Peptide scale count | DeSeq2 normalization | UniProt Homo sapiens |

**References**

1 Li, J. *et al.* TCPA: a resource for cancer functional proteomics data. *Nat Methods* **10**, 1046-1047, doi:10.1038/Nmeth.2650 (2013).

2 Cancer Genome Atlas, N. Comprehensive molecular portraits of human breast tumours. *Nature* **490**, 61-70, doi:10.1038/nature11412 (2012).

3 Liu, N. Q. *et al.* Comparative proteome analysis revealing an 11-protein signature for aggressive triple-negative breast cancer. *Journal of the National Cancer Institute* **106**, djt376, doi:10.1093/jnci/djt376 (2014).

4 De Marchi, T. *et al.* 4-protein signature predicting tamoxifen treatment outcome in recurrent breast cancer. *Molecular oncology* **10**, 24-39, doi:10.1016/j.molonc.2015.07.004 (2016).

5 Tang, W. *et al.* Integrated proteotranscriptomics of breast cancer reveals globally increased protein-mRNA concordance associated with subtypes and survival. *Genome medicine* **10**, 94, doi:10.1186/s13073-018-0602-x (2018).
