# Supplementary material for: Survival analysis in breast cancer using proteomic data from four independent datasets
Source: Sci Rep. 2021 Aug 18;11:16787. doi: 10.1038/s41598-021-96340-5 (PMC8373859; doi:10.1038/s41598-021-96340-5)
Supplement: Supplementary file 4 — Supplementary Information 4. [file 41598_2021_96340_MOESM4_ESM.docx]

**SUPPLEMENTARY MATERIAL**

**Supplemental Table 1.** Processing of proteomic data in the four protein datasets eligible for our analysis

**Supplemental Table 2:** Protein list: the final integrated table of all annotated proteins in the database, including the gene symbol, UniProt ID and TCPA antibody list.

**Supplemental Table 3:** Protein biomarkers: the list of previously published biomarker candidates related to survival.
